# Shape Selectivity of Middle Superior Temporal Sulcus Body Patch Neurons

**DOI:** 10.1523/ENEURO.0113-17.2017

**Published:** 2017-06-27

**Authors:** Ioannis Kalfas, Satwant Kumar, Rufin Vogels

**Affiliations:** Laboratorium voor Neuro- en Psychofysiologie, Department of Neurosciences, KU Leuven, Leuven, Belgium

**Keywords:** body patch, convolutional neural networks, inferior temporal cortex, macaque, object recognition, shape selectivity

## Abstract

Functional MRI studies in primates have demonstrated cortical regions that are strongly activated by visual images of bodies. The presence of such body patches in macaques allows characterization of the stimulus selectivity of their single neurons. Middle superior temporal sulcus body (MSB) patch neurons showed similar stimulus selectivity for natural, shaded, and textured images compared with their silhouettes, suggesting that shape is an important determinant of MSB responses. Here, we examined and modeled the shape selectivity of single MSB neurons. We measured the responses of single MSB neurons to a variety of shapes producing a wide range of responses. We used an adaptive stimulus sampling procedure, selecting and modifying shapes based on the responses of the neuron. Forty percent of shapes that produced the maximal response were rated by humans as animal-like, but the top shape of many MSB neurons was not judged as resembling a body. We fitted the shape selectivity of MSB neurons with a model that parameterizes shapes in terms of curvature and orientation of contour segments, with a pixel-based model, and with layers of units of convolutional neural networks (CNNs). The deep convolutional layers of CNNs provided the best goodness-of-fit, with a median explained explainable variance of the neurons’ responses of 77%. The goodness-of-fit increased along the convolutional layers’ hierarchy but was lower for the fully connected layers. Together with demonstrating the successful modeling of single unit shape selectivity with deep CNNs, the data suggest that semantic or category knowledge determines only slightly the single MSB neuron’s shape selectivity.

## Significance Statement

Functional MRI studies have shown regions in the temporal cortex that are selectively activated by bodies. In agreement with the fact that animals can be identified from their silhouette, recording studies showed that the stimulus selectivity of single units of the middle superior temporal sulcus body patch (MSB) is strongly determined by shape. Using adaptive stimulus sampling, we examined for the first time the shape selectivity of single MSB neurons, which produced a wide range of responses to a variety of shapes. Deeper layers of deep convolutional neural networks provided excellent models of the observed shape selectivity of single neurons in the fMRI-defined MSB. Overall, the data suggest that semantic or category knowledge determines only slightly the single MSB neuron’s shape selectivity.

## Introduction

Functional MRI (fMRI) studies in primates have shown cortical regions that are activated more strongly by bodies compared with other object categories (Downing et al., 2001; Tsao et al., 2003; Pinsk et al., 2005, 2009; Bell et al., 2009; Popivanov et al., 2012; Lafer-Sousa and Conway, 2013; Premereur et al., 2016). In macaques, three such body patches have been identified, and at least two located in the Superior Temporal Sulcus (STS) are functionally connected (Premereur et al., 2016). Single neurons of the middle STS body (MSB) patch show, on average, a greater response to bodies compared with images of other categories (Popivanov et al., 2014). The MSB neurons show a marked within-body category selectivity, responding only to some images of bodies, and some MSB neurons respond also to images of objects (Popivanov et al., 2014, 2016).

Recently, Popivanov et al. (2016) attempted to define the features that MSB neurons respond to by randomly occluding parts of the image (“bubbles”) that effectively drove the neuron. That study suggested that the majority of MSB neurons respond to body image fragments such as extremities or torso parts. However, characterizing the stimulus selectivity of an MSB neuron by reducing a single image to fragments has limitations, because the selectivity likely results from hierarchical nonlinear processing (DiCarlo et al., 2012), and thus cannot be fully characterized by “effective” fragments of a single image.

Here, we examined the selectivity of MSB neurons not by identifying critical features that drive the response to a single image, but by measuring and modeling their response to a large variety of stimuli. To reduce image space, we examined shape selectivity, measuring the responses of MSB neurons to silhouettes of the original shaded and textured stimuli. Previous work found that MSB neurons show similar selectivity for silhouettes and the original images (Popivanov et al., 2015), suggesting that the shape selectivity is a strong determinant of the overall stimulus selectivity of MSB neurons.

Our previous single unit studies suggest that MSB neurons respond to only a small portion of the vast shape space. Given such high degree of shape selectivity, presenting a fixed set of randomly chosen shapes will likely undersample the portions of shape space in which shape variations produce marked differences in response. Furthermore, such relevant portions of shape space will differ between neurons. Thus, to have a more efficient exploration of relevant portions of shape space for each neuron, we used an adaptive stimulus sampling strategy (Yamane et al., 2008; Okazawa et al., 2015). We modified the shape of silhouettes to which the neuron responded, presented these modified shapes together with randomly selected shapes, and measured the response of the neuron to those stimuli. Then, we again modified shapes that elicited a response from the neuron. This adaptive procedure of response-based shape selection and modification followed by measurement of the responses to the newly generated shapes was repeated in successive iterations, resulting in a wide range of responses of the neuron for a variety of shapes.

Although previous studies of body patch neurons showed, on average, greater responses to bodies compared with other stimulus categories, those studies used a relatively small number of stimuli, providing a limited description of their stimulus selectivity and effective stimuli. Here we used a much larger set of shapes, adapted to the individual neurons’ response, which allowed us to examine whether their effective stimuli resemble bodies or instead shapes that are not bodies. Furthermore, we aimed to provide a predictive model of MSB neuron’s shape selectivity. A previous study described the shape selectivity of posterior inferotemporal (IT) neurons (Brincat and Connor, 2004) by a model that parameterizes shapes in terms of curvature and orientation of local contour segments. Thus, we examined to what degree such a model can describe the shape selectivity of MSB neurons. The performance of this model was compared with that of a pixel-based gray-level model and multilayered convolutional neural networks (CNN), including deep neural networks (Krizhevsky et al., 2012; Kriegeskorte, 2015). Deep CNNs, trained with natural images for classification, have been shown to outperform other models (e.g., HMAX; Riesenhuber and Poggio, 1999) in predicting the stimulus selectivity of IT multiunit activity (Cadieu et al., 2014). However, it is unknown how well these deep neural networks model shape selectivity of single neurons of an fMRI-defined body patch. To address this, we fitted the shape selectivity of MSB neurons with a linear weighting of the activations for each deep neural network layer and compared the fits across layers.

## Materials and Methods

### Single unit recordings

#### Subjects

We used the same two male rhesus monkeys (*Macaca mulatta*) as in our previous studies of MSB (Popivanov et al., 2014, 2015, 2016). They were implanted with a headpost and a recording chamber targeting MSB. Animal care and experimental procedures complied with national and European laws, and the study was approved by the Ethical Committee of the Katholieke Universiteit Leuven.

#### Recordings

We recorded from the left MSB, as defined by fMRI in the same subjects by contrasting images of headless monkey bodies and control objects (for details, see Popivanov et al. [2014]). For each guide tube position targeting the fMRI-defined body patch, we verified that MSB neurons responded to the images of monkey bodies, human bodies, mammals, or birds that were used in the fMRI study of Popivanov et al. (2014). Single unit recordings were performed with Epoxylite-insulated tungsten microelectrodes (FHC; *in situ* measured impedance 0.8–1.7 MΩ) using techniques described previously (Popivanov et al., 2014). Briefly, the electrode was lowered with a Narishige microdrive into the brain using a guide tube that was fixed in a standard Crist grid positioned within the recording chamber. After amplification and filtering between 540 Hz and 6 kHz, single units were isolated online using a custom amplitude- and time-based discriminator.

The position of one eye was continuously tracked by means of an infrared video-based tracking system (SR Research EyeLink; sampling rate 1 kHz). Stimuli were presented on a CRT display (Philips Brilliance 202 P4; 1024 × 768 screen resolution; 75 Hz vertical refresh rate) at a distance of 57 cm from the monkey’s eyes. The onset and offset of the stimulus were signaled by means of a photodiode detecting luminance changes of a square in the corner of the display that was invisible to the animal. A digital signal processing–based computer system controlled stimulus presentation, event timing, and juice delivery while sampling the photodiode signal, eye positions, spikes, and behavioral events. Time stamps of the spikes, eye positions, stimulus, and behavioral events were stored for offline analyses.

#### Stimuli

**Figure 1.**
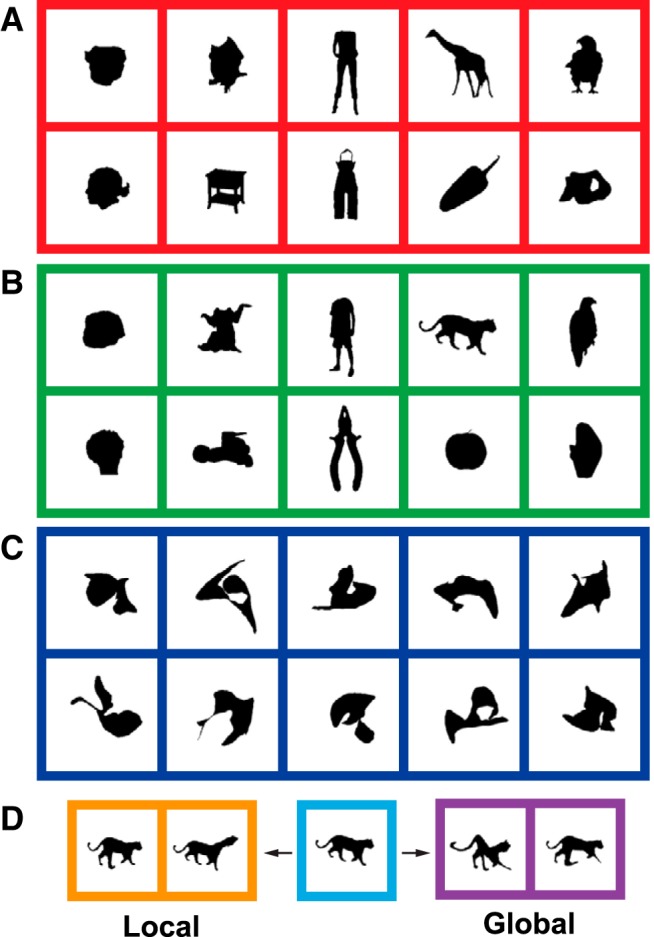
Examples of stimuli. ***A***, ***B***, Examples of silhouettes of the two stimulus sets used during the search task. ***C***, Examples of “random shapes” used in adaptive stimulus sampling test. ***D***, Examples of two local and two global shape deformations. The parent image is shown in the middle panel.

Responsive MSB neurons were searched with two fixed sets of 100 stimuli each. Both search sets included silhouettes of 10 monkey bodies and 10 human bodies (excluding the head), 10 monkey faces and 10 human faces, 10 four-legged mammals (with head), 10 birds (with head), 10 fruits/vegetables, 10 body-like sculptures, and 20 human-made objects. The first set of 100 silhouettes corresponded to the same images used by Popivanov et al. (2015) and were used in 79 of 100 tested neurons. The remaining 21 neurons were tested with silhouettes of the “odd” images of Popivanov et al., (2012). The silhouettes were resized so that their area equated that of a circle of 4° diameter (12.56 deg^2^). The silhouettes had black pixels (0.01 cd/m^2^) and were presented on a uniform colored background (54 cd/m^2^; R 230, G 230, B 250). Examples of stimuli of each search set that were used to find responsive neurons are presented in Fig. 1*A*, *B*.

For the adaptive stimulus sampling procedure, novel shapes were created online. To do so, we parameterized the contours of the shapes with elliptical Fourier coefficients (Kuhl and Giardina, 1982) using 128 harmonics. This number of harmonics was sufficient to obtain an approximation of the silhouette of a body or object that was perceptually similar to its original. Note that we used this parameterization as a computationally efficient and fast tool to manipulate and generate a wide variety of shapes, but we do not assume or imply that the neurons encode elliptical Fourier features. The novel shapes either were derived from the silhouettes of the search set (see below) or were randomly generated. The latter we label “random shapes,” since they occupy a random position in shape space. The random shapes were created by randomizing each of the four coefficients of the 128 harmonics. The range of the values for each of the harmonics was constrained to decrease monotonically, so that lower harmonics were allowed to have a larger amplitude than the higher harmonics. This was done to ensure that the frequency spectrum of the random shapes and the body silhouettes were similar. Examples of random shapes are shown in Fig. 1*C*.

We also derived novel shapes from stimuli, which we label “parent stimuli,” shown during the search test or during earlier generations of the adaptive stimulus sampling procedure (see below). We generated four novel shapes from each parent stimulus, two with “local” shape deformations and two with “global” shape deformations. First, we put a 3 × 3 rectilinear grid (4 × 4 vertices) on top of the contour of the parent shape. The outer dimensions of this transformation grid equaled the bounding box of the shape. A local deformation consisted of a random displacement (within an arbitrarily chosen range) of one randomly chosen vertex of the grid, whereas a global deformation consisted of random displacements of each of five different randomly chosen vertices. The initial shape was then deformed according to the transformation grid, using cubic interpolation. The novel shape generated was then approximated by 128 elliptical Fourier harmonics. Examples of such deformed shapes are shown in Fig. 1*D*.

The outer contour of all novel shapes was required to be closed while avoiding any self-intersections. Furthermore, their maximal extent was constrained to be 12° while maintaining their area equal to 12.56 deg^2^. The mean width and height of the shapes was 5.3° and 6°, respectively. The stimuli were shown centrally, except in a minority of neurons that failed to show sizable responses to foveal presented stimuli, in which we presented the stimuli eccentrically (based on receptive field mapping). The silhouettes were centered according to their center of mass.

#### Tests

In all tests, stimuli were presented for 200 ms each, with an interstimulus interval of at least 300 ms during passive fixation (fixation window size 2° × 2°). Fixation was required in a period from 100 ms prestimulus to 200 ms poststimulus. Only unaborted trials were taken into account. Juice rewards were given with decreasing intervals (exact values depending on the animal) as long as the animal maintained fixation. Reward delivery was not locked to the stimulus presentations.

### Search test

Neurons were tested with pseudorandom, interleaved presentations of the 100 silhouettes of either set (Popivanov et al., 2014). The number of unaborted presentations per stimulus was three. Based on this test, stimuli were selected for subsequent tests. In some neurons, only weak responses were present for foveally presented stimuli. For these neurons (*n* = 16), we performed receptive field mapping (Popivanov et al., 2015), followed by repetition of the search test with presentation of the stimuli at the hotspot of the receptive field.

### Adaptive stimulus sampling test

This test was run automatically online with a Matlab-based program. Firing rates were computed for each trial using a window whose poststimulus onset ranged from 50 to 250 ms and were then averaged across presentations per stimulus. After finishing the search test, the program selected those stimuli that produced the five greatest mean firing rates. These five stimuli became the parent stimuli of the first generation of the adaptive stimulus sampling test. We used two adaptive stimulus sampling procedures.

The first procedure was run on 26 neurons. In this procedure, four novel shapes were derived of each parent stimulus, totaling 20 new shapes. In addition, we generated 25 random shapes. These 45 stimuli constituted the first-generation stimuli. The next generations of stimuli were defined as follows. The eight images that produced the greatest firing rates of all stimuli of all preceding generations were selected, and four novel shapes were derived for each of them. These 32 novel shapes, together with eight novel random shapes and five shapes of the preceding generations, constituted the 45 stimuli of the next generation.

The second stimulus sampling procedure aimed to sample a more uniform spread of responses than the first procedure. It had the same stimulus selection of first-generation stimuli as the first procedure. However, for the next generations, we used a different stimulus selection procedure. We binned the stimuli of the preceding generations into five bins based on their firing rate. The width of each bin corresponded to 20% of the maximal firing rate of the neuron, computed across all tested stimuli. Thus, the first bin contained those images with a firing rate 81–100% of the top image firing rate, the second bin contained images with 61–80% of the top image firing rate, and so on. Then, we sampled at random three stimuli from the first bin (81–100%), two stimuli from the second bin (61–80%), two stimuli from the third bin (41–60%) and one stimulus from the fourth bin (21–40%). In the case that a particular bin did not have a sufficient number of images (e.g., only 2 images in the first bin), the remaining images were taken from the next bin (e.g., one extra image taken from the second bin). Then, we derived four novel shapes from each of the eight selected stimuli. These 32 novel shapes, together with eight novel random shapes and five shapes of preceding generations, constituted the 45 stimuli of the next generation. The second procedure was performed for the majority of neurons (*n* = 51).

In both procedures, the test of each generation of stimuli included five unaborted presentations of each stimulus. The stimuli were presented in pseudorandomized order, with the same reinforcement schedule as in the search test. We ran the adaptive stimulus sampling test as long as we could hold the neuron or had a large number of generations. In the present study, we included only the 77 neurons for which at least five generations were available. The mean number of generations was 9.5 (range, 5–20), which compares well with previous studies using adaptive stimulus sampling (Yamane et al., 2008; Okazawa et al., 2015).

### Shape decomposition test

In this test, we examined the responses to different parts of the top shape of the adaptive sampling test, i.e., the shape that produced the greatest response in that test. Popivanov et al. (2016) showed that single MSB neurons are sensitive to removal of some parts of an effective shape. Thus, this part manipulation provides an additional set of stimuli to which we measured responses and that could be used as an independent test of the shape selectivity models. We conducted the shape decomposition test in the small number of neurons that we were able to record after finishing the adaptive stimulus sampling test. We selected the shape that produced the greatest response across all the stimuli of all generations of the adaptive stimulus sampling test. Then we removed parts of this top shape, e.g., a limb-like feature, a head-like feature, or part of the torso. The stimulus reduction was performed online by the experimenter, using a custom-made graphical interface. Different shape parts could be presented in different tests, with each test always including the full top shape. For instance, a test could include presentations of only the left arm, only the right arm, the body-like shape without both arms, and the full top shape. A next test for the same neuron then consisted of, for example, presentations of the half of the torso, the legs, and the full top shape. The parts were presented at the same size and at the same location as in the top image. In some neurons, we also included rotations (90°, 180°, or 270°) of the top shape. Stimuli were presented in pseudorandomized order, using the same presentation and reward schedules as in the other tests. We included in the analysis only those tests with at least seven unaborted presentations per stimulus.

### Data analysis

We used raw firing rates, unless otherwise stated. In the case of analyses of net firing rates, we subtracted the firing rate in a baseline window ranging from 100 to 0 ms before stimulus onset from the raw firing rate (computed in a response window ranging from 50 to 250 ms after stimulus onset; see above).

The degree of body-category selectivity of a single neuron was quantified by the body selectivity index (BSI; Popivanov et al., 2014):

$$BSI=\frac{{\overset{¯}{R}}_{body}-{\overset{¯}{R}}_{nonbody}}{\left|{\overset{¯}{R}}_{body}\right|+\left|{\overset{¯}{R}}_{nonbody}\right|},$$

where ${\bar{R}}_{body}$ and ${\bar{R}}_{nonbody}$ are the mean net firing rates to silhouettes of bodies (monkey bodies, human bodies, mammals, and birds) and nonbodies (faces, objects, fruits/vegetables) of the search stimulus set, respectively.

The Spearman–Brown corrected split-half correlation coefficient *r_sh_* served as a metric of the reliability of the responses of a neuron to the stimuli of the adaptive stimulus sampling test. To compute *r_sh_*, we used all presentations of all stimuli presented in the adaptive sampling test. The explainable variance of a neuron was defined as the *r_sh_*^2^.

### Human psychophysics

The psychophysical data were obtained using Amazon’s Mechanical Turk crowdsourcing platform. For experimental design and stimulus presentation, we used Psiturk (Gureckis et al., 2016), an open-source framework for conducting behavioral experiments online, and JsPsych (de Leeuw, 2015), a JavaScript library for creating behavioral experiments in a Web browser. The task was performed by 55 human subjects, recruited from the MTurk subject pool, for a small fee.

We asked the subjects to rate the top and bottom shapes, defined for each neuron by the maximal and minimal firing rate, respectively, in the adaptive stimulus sampling test. The instruction to the subjects was as follows: “You will have to rate a set of black and white silhouettes of pictures. We wish to know the degree to which you feel that the picture can be of a living creature, an animal or a human(oid). Note that some body parts may be missing (like the head) or may be exaggerated in their form or size. You have to rate each silhouette using a scale that ranges from 1 to 5 with the extremes of the scale corresponding to 1, very much animal-like, and 5, not at all animal-like. By animal-like, we mean animals and humans. The animals do not have to correspond to a real animal living on our planet—judge whether they look like an animal.” A trial started with the onset of a small red fixation target in the middle of the display for 1000 ms, followed by a 200-ms-long presentation of the black shape with the fixation target on top of it. The background was white. After the stimulus presentation, we presented the empty white background for 500 ms, after which a horizontal rating scale, labeled as in the instructions, was shown. The subjects had to indicate their choice by pressing one of the five numbers on the keyboard. After responding, the next trial started. We computed the median rating across subjects for each stimulus. *r_sh_* was 0.93 (*n* = 154 stimuli), suggesting that we had a sufficient number of subjects to have a reliable estimate of the rating scores of humans.

### Modeling

#### Curvature and angular position tuning models

The curvature and angular position tuning (CAP) family of models was adapted from Brincat and Connor (2004). In these models, the neuron summates the output of a small number of subunits, each tuned for a combination of curvature, orientation, and position (*x* and *y* coordinates) of contour elements. The summation can be exclusively linear or also include nonlinear terms. Furthermore, the subunits can be exclusively excitatory or both excitatory and inhibitory. Thus, we distinguished four types of models: the standard model that includes both excitatory and inhibitory subunits and a nonlinear summation term (E-I-NL model), one that includes only excitatory subunits and a nonlinear term (E-NL model), one that includes both excitatory and inhibitory subunits but no nonlinear term (E-I model), and one that includes only excitatory subunits and no nonlinear term (E model). Below we briefly describe our implementation of this family of models and refer to Brincat and Connor (2004) and Pasupathy and Connor (2001) for more details. Note that Brincat and Connor (2004) considered only the E-I-NL model, which was also our standard CAP model.

First, the outer contour of each shape was represented by elliptical Fourier coefficients of the first 24 harmonics. This smoothened pixelated edges for reliable estimation of curvature. The curvature was defined as the rate of change in tangent angle with respect to contour length. It was estimated using the same procedure as Pasupathy and Connor (2001), which computes absolute and not relative curvature (El-Shamayleh and Pasupathy, 2016). Next, the contour was decomposed into elements of approximately constant curvature. To avoid extreme curvature values, we used the following squashing function (Pasupathy and Connor, 2001):

$$\kappa^{\prime }=\frac{2.0}{1+e^{-c\kappa }}-1.0$$

where κ and $\kappa^{\prime }$ are the curvatures before and after the squashing transformation, respectively. The squashing function slope c was set to 20. The orientation (θ) of the contour element was defined as the polar angle of the perpendicular bisector that points outward from the interior of the silhouette. The position of the contour element, in *x* and *y* coordinates, was defined as the bisector point of the line joining the end points of the contour element.

We modeled each cell’s stimulus selectivity with a combination of four-dimensional (4D) Gaussian subunits. The dimensions were curvature κ, orientation θ, and *x* and *y* position. Our standard model (E-I-NL) is the same as that of Brincat and Connor (2004), except in our case the relative and absolute position coordinates of the contour elements were identical (unlike in Brincat and Connor [2004], our stimuli were centered according to their center of mass), so their 6D model was reduced to a 4D model. The Gaussian subunit $G_{s}$ was defined by the following equation:

$$G_{s}=\omega_{s}\cdot \overset{\#\text{contour elements}}{\underset{n=1}{\sum }}e^{-\left[\frac{{\left(\kappa_{n}-\mu_{k}\right)}^{2}}{2\sigma_{\kappa }^{2}}+\frac{{\left(\theta_{n}-\mu_{\theta }\right)}^{2}}{2\sigma_{\theta }^{2}}+\frac{{\left(x_{n}-\mu_{x}\right)}^{2}}{2\sigma_{xy}^{2}}+\frac{{\left(y_{n}-\mu_{y}\right)}^{2}}{2\sigma_{xy}^{2}}\right]}$$

where κ, θ, *x* and *y* are the curvature, orientation, and position coordinates, respectively, of contour element *n*; µ and σ correspond to the fitted mean and SDs for each of the four Gaussian dimensions; and ω*_s_* is the fitted amplitude of the subunit. As in Brincat and Connor (2004), the SD of the *x* and *y* position coordinates (σ*_xy_*) was constrained to be identical.

The models could have one to six Gaussian subunits. In the standard model, ω*_s_* can have negative or positive values, corresponding to an inhibitory or excitatory subunit, respectively. The response predicted by that model is the sum of (a) the summed responses of the individual subunits, each weighted by their amplitude weight ω*_s_*, and (b) the weighted product of the same subunits of which ω*_s_* has the same sign. Fitted parameters ω*_NL+_* and ω*_NL–_* describe the weights of the products of the excitatory and inhibitory subunits, respectively. Thus,

$$\text{E-I-NL}=g{\left\lfloor \sum_{s=1}^{\#\text{subunits}}\left(G_{s}\right)+\omega_{NL+}\prod_{s=1}^{\#\text{excitatory subunits}}\left(G_{s}\right)+\omega_{NL-}\prod_{s=1}^{\#\text{inhibitory subunits}}\left(G_{s}\right)\right\rfloor }^{+}+b_{0},$$

where $b_{0}$ is the fitted baseline firing rate, *g* is the fitted gain, and the brackets represent half wave rectification of the response. The standard model had the largest number of fitted parameters compared with the other three models of this family. The number of fitted parameters depended on the number of subunits and was 9, 16, 21, 27, 32, and 37 for 1 to 6 subunits in the standard model, respectively. The E-NL model differed from the standard model by constraining ω*_s_* to be positive. The E-I and E models did not contain nonlinear terms. The E model differed from the E-I model by having positive ω*_s_* values only.

The model parameter search space was constrained as follows. The ranges of the means of the Gaussian subunits were restricted to their physically possible range (e.g., *x* and *y* coordinates within the union of all presented shapes). As in Brincat and Connor (2004), the SD for a particular dimension was equal across all subunits. The standard deviation (σ) of the subunits were constrained to be within a physiologically plausible range for each dimension. For curvature, the bounds for the SD were 0.01–0.5. Similarly, SDs for orientation and position were bounded within a range of 7.5–90° and 20 to (1/3) × (mean of maximum extent for all shapes across the *x* and *y* dimensions) range, respectively. As in Brincat and Connor (2004), the means of the Gaussian subunits were constrained to be separated by at least 2SDs in the 4D space, so that the subunits were sufficiently different. We implemented this constraint in the model evaluation function by calculating whether the current parameter estimate included any pair of subunits with less than the stated separation criterion. If so, the calculated model residuals were multiplied by a penalty value of 5, to effectively prevent the solver function from exploring those parts of the parameter space. Following Brincat and Connor (2004), we also constrained the sum of all positive ω*_s_* and ω*_NL+_* to be 1.5 times the maximum observed firing rate as an upper bound, and the sum of all the negative ω*_s_* and ω*_NL–_* was constrained to be –1.5 times the maximum firing rate as a lower bound. This ensured that the fitted weights and predicted responses stayed within a realistic range across the entire shape domain.

Models were fitted for each neuron using the extracted curvature, orientation, and position coordinate values of the contour segments of each of the shapes. Each neuron’s dataset was split randomly into five nonoverlapping subsets with each subset containing 20% of the data. We used a fivefold cross-validation approach to counter overfitting. In this approach, a single subset of the five data subsets was retained for testing the model, and the remaining four subsets were used as training data. This process was repeated five times, and each of the five subsets was used exactly once as test data. An iterative nonlinear least-squares algorithm (Matlab, *lsqnonlin* function with *trust-region-reflective* method) with an objective to minimize the sum of squared differences between observed and predicted responses was implemented for fitting the models. The implementation of constraints (see above) prevented the solver function from exploring certain parts of the parameter space, rendering the error surface discontinuous. Therefore, we fitted models from different starting points, which avoided getting stuck in local minima and produced consistent and nondegenerate models. To do this, we used the *multistart* algorithm, implemented in Matlab, for model optimization to find a global minimum from multiple local minima. Models with *n* subunits were instantiated with *n* × 100 random starting points (within the bound constraints) to sample the parameter space in a dense manner.

For each of the five cross-validation runs, we computed the Pearson correlation coefficient *r* between the predicted and measured responses for the independent 20% test data. The mean of the five correlation coefficients was taken as a goodness-of-fit metric. Because the goodness-of-fit cannot exceed the reliability of the data, we also computed the explained explainable variance by dividing the mean explained variance by the model (mean *r*^2^ of the five cross-validated runs) by the explainable variance (*r_sh_*^2^) of the responses of the neuron in the adaptive stimulus sampling test.

#### Pixel gray-level model

This is a simple model that consists of a linear combination of the weighted-pixel gray-levels of the shapes. The input vector for a particular image consists of the concatenated rows of the gray levels of that image. Each pixel value is multiplied by a weight followed by summation of the weighted values. For each neuron, we applied partial least squares (PLS) regression (Wold et al., 1984) to estimate the weights for individual pixels. PLS constructs new predictor variables, known as components, as linear combinations of the original predictor variables. The components are found using the covariance of the predictor input matrix X (here, pixel gray values for the shapes tested in a neuron) and the correlation between X and the response vector Y (here, responses of that neuron to the shapes). PLS thus combines the information regarding the variances of X and Y while also taking into account the correlations among them. The PLS regression procedure we adopted was as follows. We assigned randomly 80% of the data to a training set, and the remaining 20% constituted the test set. We performed PLS regression (using SIMPLS in Matlab) with up to 30 components and fivefold cross-validation on the training data set. For each neuron, we defined the optimal number of components by the global minimum of the mean squared error function. Then, we ran PLS with the selected number of components, followed by testing the model on the 20% held-out data. Goodness-of-fit and explained explainable variance were computed in the same way as for the CAP models. We ran the same PLS procedure on surrogate data in which the assignment of mean neural response to stimulus was randomized. The models trained on the surrogate data performed very poorly, explaining only 1% of the response variance (median *r*^2^ = 0.01). This demonstrates the validity of the PLS procedure.

#### HMAX models

We used the implementation of the shallow convolutional network HMAX by Mutch and Lowe (2006). For each stimulus, we computed the activations of C1 units. The C1 layer performs a max pooling operation on the output of the S1 orientation selective filters of the preceding layer, producing some degree of position and size tolerance. The number of C1 units for each of four orientations (step = 45°) depended on scale, ranging from 25 (5 × 5; coarse scale) to 625 (25 × 25; fine scale). Each of eight C1 scales could define a different model. For each scale, the C1 activation vector for the shapes, which included unit activations for all four orientations (e.g., vector length = 4 × 25 × 25), served as predictor of the responses of a neuron. We used the same PLS regression procedure (with fivefold cross-validation and independent train and test data) as described above for the pixel gray-level model to predict the responses of individual neurons from the HMAX C1 activations for each scale.

#### Alexnet

This deep convolutional neural network, developed by Krizhevsky et al. (2012), consists of eight weight layers: five convolutional layers followed by three fully connected layers. Some of the first five layers consist of three stages: convolution, max pooling, and normalization. We used the version of Alexnet trained to classify ∼1.2 million natural images divided in 1000 classes for the ImageNet Large Scale Visual Recognition Challenge 2012 (ILSVRC2012). For each stimulus, we computed the activations of all the units of each stage of the layers, including the pooling and normalization stages, using feature extraction from the Matconvnet Matlab toolbox. The input to Alexnet was the shape image minus the mean of the ILSVRC2012 training images, since this subtraction from the mean was also performed when training Alexnet. Then, we performed PLS regression to predict the responses of a single neuron from the activations of a layer stage to the stimuli as predictor. The PLS regression procedure was identical to the one described above. For each neuron, Alexnet yielded a separate model per layer stage, which allowed comparison of the explained explainable variances across layers.

#### Vgg-19

This very deep convolutional neural network by Simonyan and Zisserman (2014) consists of 19 weight layers: 16 convolutional layers and three fully connected layers. In addition, it has five max pooling stages in between the main convolutional layers. We used the trained version of VGG-19 that is available from Matconvnet, which was also trained to perform classification on the ILSVRC2012 data. As with Alexnet, we first subtracted the mean of all trained images from the shape stimulus. We used the same PLS regression procedure as with Alexnet to predict the responses of the single neurons from the VGG-19 layer activations.

## Results

**Figure 2.**
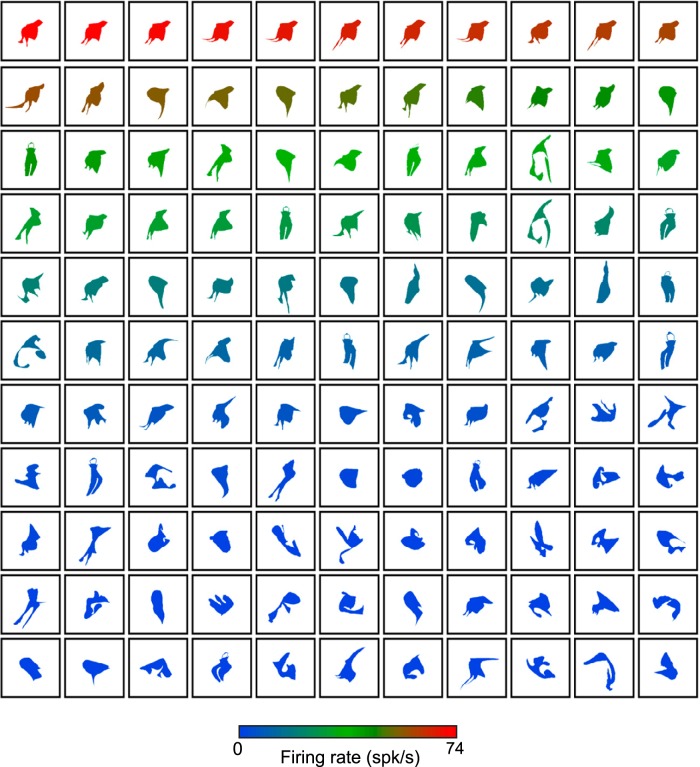
Responses of an example neuron in the adaptive stimulus sampling test. Neuron C65b was tested with 605 silhouettes. Every fifth shape is plotted and ranked according to firing rate, which is indicated by the color of the shape.

**Figure 3.**
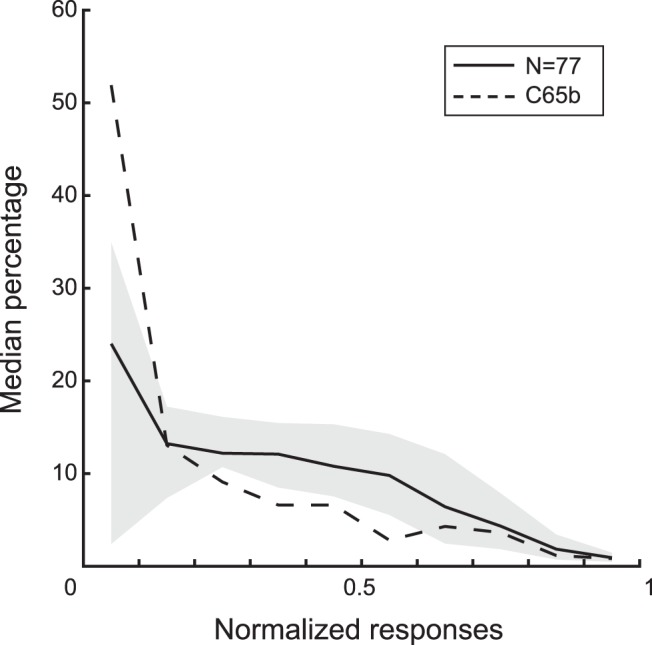
Distribution of responses in the adaptive stimulus sampling test. For each neuron, we binned the normalized responses in 10 bins (bin width 0.1) and then computed the percentage of stimuli per bin. The responses were normalized by the maximum response of the neuron. The full line shows the median percentage of stimuli for each response bin, across the 77 neurons. The band corresponds to the interquartile range. The stippled line shows the percentage of stimuli for the different response bins for the example neuron C65b.

We recorded the responses of 100 single MSB neurons of two monkeys (monkey E, 63 neurons; monkey B, 37 neurons) to silhouettes of 100 images of various categories, including bodies, faces, and human-made objects. After this search test, we were able to measure the responses of 77 MSB neurons (monkey B, 29 neurons) in the adaptive stimulus sampling test. These MSB neurons responded on average more strongly to silhouettes of bodies of monkeys, humans, animals, and birds compared with silhouettes of other categories. The median BSI in the search test for the 77 neurons was 0.19, which was significantly greater than 0 (Wilcoxon test, *p* < 0.001), with 42% of the neurons having a BSI >0.33, i.e., twofold stronger responses to bodies, compared with object and face silhouettes. In the adaptive sampling test, we modified the shape of silhouettes that were selected based on the firing rate of the neuron to the stimuli. As described in detail in Materials and Methods, we selected the five images that produced the greatest firing rate in the search test and then modified their shape, producing 20 novel shapes. These modified shapes were presented interleaved with 25 randomly generated shapes (“random shapes”), and the responses to these 45 stimuli were recorded. In successive generations, we selected eight shapes that produced high firing rates in previous generations and modified their shape. In each generation, the new modified shapes were presented interleaved with new random shapes. This adaptive stimulus sampling procedure produced a wide range of responses for a large variety of shapes, which is illustrated for an example neuron C65b in Fig. 2. This neuron was tested with 605 shapes in the adaptive stimulus sampling test. The mean number of shapes tested per neuron was 358 (*n* = 77 neurons), ranging from 205 to 805. To illustrate the distribution of the responses across stimuli, we computed for each neuron the percentage of shapes in 10 nonoverlapping bins, each having a width of one tenth of the maximal firing rate of that neuron. For the 77 neurons tested with the adaptive stimulus sampling procedure, the median proportion of shapes decreased monotonically with increasing response strength (Fig. 3), demonstrating the strong shape selectivity of the MSB neurons.

**Figure 4.**
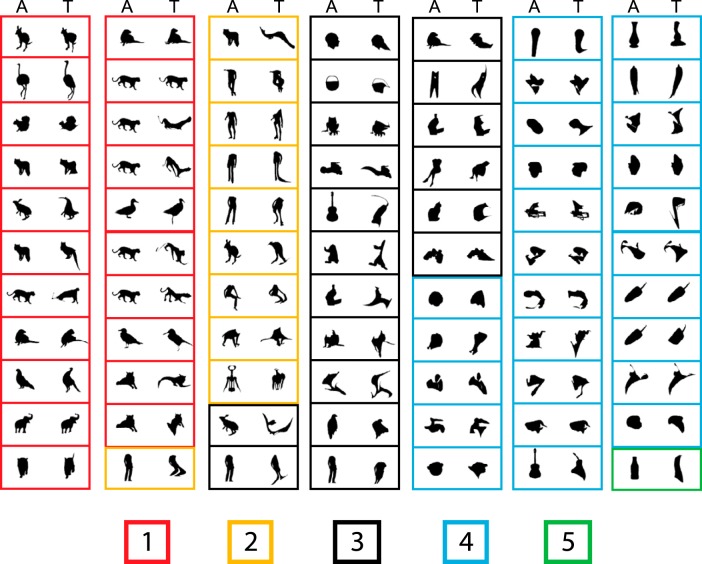
Top images of all neurons (*n* = 77) obtained with the adaptive sampling test. In each panel, the right image corresponds to the top shape (T) of the neuron, and the left image is the ancestor image (A) from which the top image was derived. Each panel corresponds to a single neuron. The panels are grouped according to the median rating of the top image in the human psychophysical study, ranging from 1, very much animal-like, to 5, not at all animal-like. The rating is indicated by the color of the panel’s frame according to the legend at the bottom.

The adaptive stimulus sampling procedure produced stronger responses, i.e., more effective stimuli, than the search test that used a fixed set of 100 stimuli. Indeed, the median firing rate in our sample of neurons to the top image of the adaptive stimulus sampling test was 73 spikes/s (*n* = 77), which was significantly higher than the median firing rate of 33 spikes/s to the most effective stimulus of the search test (Wilcoxon signed rank test; *p* = 1.34 × 10^−7^). Fig. 4 shows for each neuron the top image of the adaptive stimulus sampling test together with the ancestor image from which it was derived across preceding generations. The ancestor images were bodies (monkey and human bodies, mammals, and birds) from the search test in 54.5% of the neurons. For the remaining neurons, the ancestor images were silhouettes of faces (6.5%), objects (15.6%), sculptures (3.9%), and fruits/vegetables (5.2%) from the search test or random shapes (14.3%) generated during the adaptive stimulus sampling test itself. It is interesting to observe that some silhouettes of nonbody ancestor images, such as a corkscrew, evolved into a more body-like shape with limb-like features.

Because MSB is defined by contrasting fMRI activations to images of (monkey) bodies with those of objects, one may expect that the top shapes of MSB neurons resemble bodies. To assess this, 55 naive human observers rated with a 5-point Likert-scale how animal-like they perceived the top images of the 77 neurons. To have a large variety of images (and ratings), we also included the bottom images. The top images shown in Fig. 4 were grouped according to their median rating. Although the top images showed a wide distribution of ratings, 40% were rated as animal-like. This shows that some MSB neurons can respond well to shapes that humans do not judge as resembling a body or an animal. Only one of the bottom images was rated as animal-like. The latter, however, should not be interpreted as suggesting that only nonbody images elicit weak responses of MSB neurons. Indeed, single MSB neurons typically respond selectively to bodies or animals, showing no or little response to particular images of bodies or animals (Popivanov et al., 2014).

**Figure 5.**
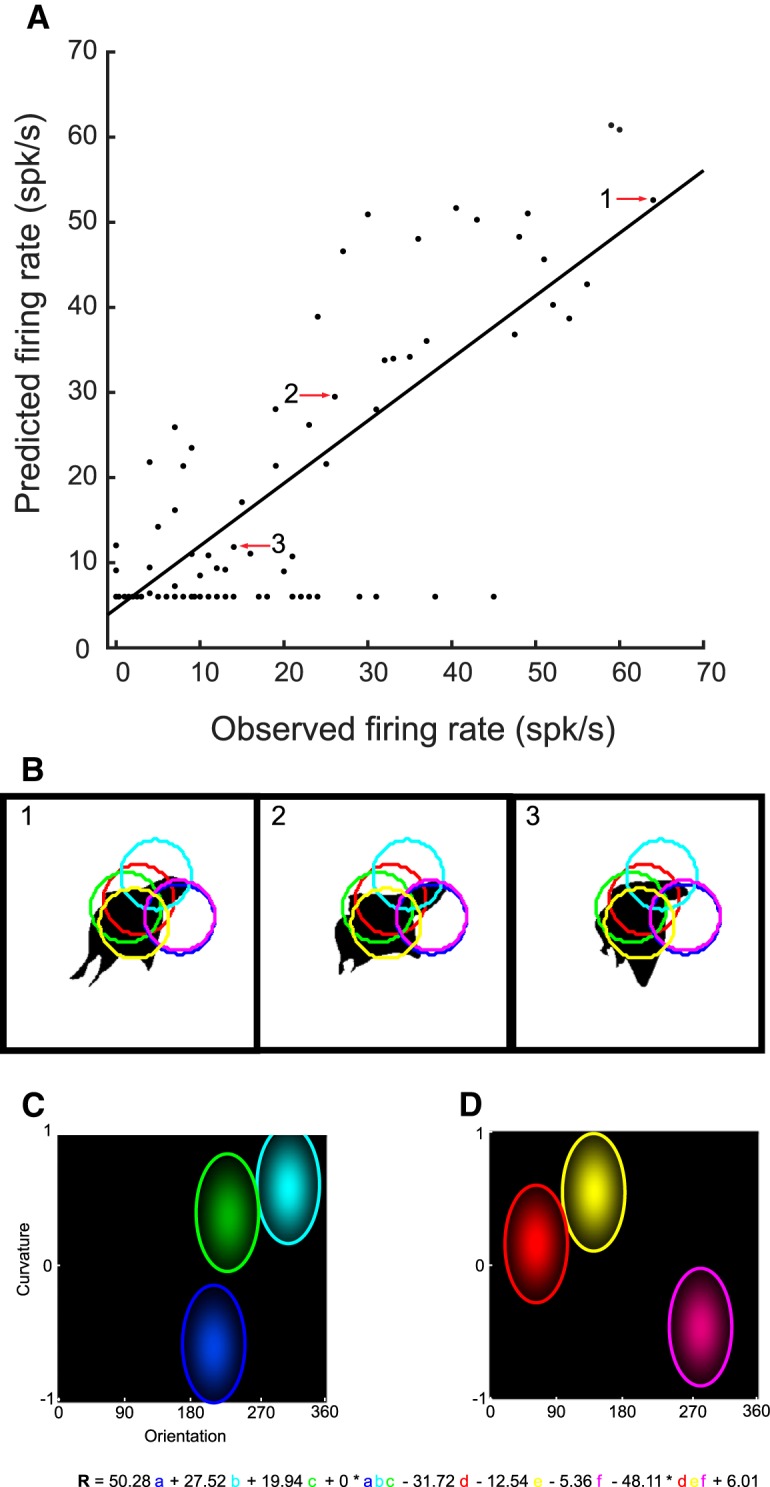
Performance of the E-I-NL CAP model with six subunits for example neuron C65b. ***A***, Scatterplot of predicted and observed firing rates for the test stimuli that were not used when fitting the E-I-NL model using six subunits. Data of one of the five cross-validation folds is shown. Full line indicates the regression line (Pearson *r* = 0.83). ***B***, Illustration of three images of which the predicted and observed responses are indicated by the arrows and numbers in ***A***. The images elicited a high (1), intermediate (2), and low (3) response, respectively. The shape selectivity of the cell was explained by six subunits the locations of which are indicated by the colored circles. The radius of the circles equals the SD of the Gaussian for the *x* and *y* dimensions. The color of the circles corresponds to that of the subunits (labeled a, b, c, d, e, and f with a–c excitatory and d–f inhibitory subunits) in the equation shown at the bottom. ***C***, ***D***, Orientation and curvature tuning of the excitatory (***C***) and inhibitory (***D***) subunits. Same conventions as in ***B***.

The shape selectivity of single MSB neurons is only partially described by the top (and bottom) images. To gain a fuller and quantitative description of the shape selectivity of an MSB neuron, we modeled the responses of each single neuron to all stimuli tested in that neuron in the adaptive stimulus sampling test. The first family of model we considered was the CAP models, adapted from Brincat and Connor (2004). In these models, a shape is formalized by the curvature, orientation, and *x* and *y* position of contour elements with an approximately constant curvature. The model neurons received input from subunits that showed Gaussian tuning in the 4D space defined by curvature, orientation, and *x* and *y* location. In the standard CAP model (Brincat and Connor, 2004), the model neuron summed the weighted responses of its subunits to contour segments and of their nonlinear interaction followed by rectification (see Materials and Methods). The input weights, tuning centers, and widths were fitted for each neuron separately with fivefold cross-validation. Models with a different number of subunits were fitted separately. The results of the model fit, with six subunits, is shown for the example neuron C65b in Fig. 5. For this neuron, the mean goodness-of-fit, averaged across the five cross-validations, computed as the correlation between observed and predicted responses for a held-out, independent sample of stimuli, was 0.78 (SD across the five folds = 0.04). The model attempted to capture the selectivity of the neuron by three excitatory and three inhibitory subunits tuned to differently oriented curved contour segments. It could predict not only weak responses to ineffective stimuli and strong responses to effective stimuli, but also responses to stimuli that produced a moderate response in the neuron (Fig. 5). However, the mean explained variance of the model (mean *r*^2^ = 0.62) was less than the explainable variance of the neuron’s responses (*r_sh_*^2^ = 0.94), resulting in a mean explained explainable variance of 0.66 (see Materials and Methods).

**Figure 6.**
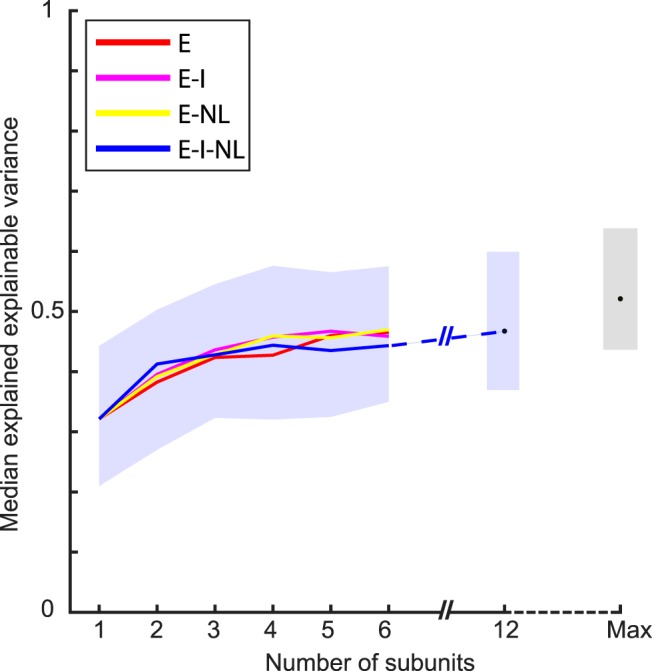
Performance of the CAP models. The median explained explainable variance (*n* = 77 neurons) are plotted for each of the four versions as a function of the number of subunits. Only the E-I-NL model version (the standard model) was fitted with 12 subunits. The blue bands indicate the interquartile ranges of the E-I-NL model version (similar for the other three versions). Max corresponds to the maximal explained explainable variance computed per neuron across the number of subunits and four model versions. The black point and gray band indicate the median and interquartile range, respectively, for the 77 neurons.

Across the 77 neurons, the median goodness-of-fit (*r*) of the standard CAP model having six subunits was 0.60 (first and third quartiles: 0.51 and 0.68). This was well below the median reliability *r_sh_* of 0.90 of the responses of the 77 neurons. The median explained explainable variance was 0.44 (quartiles, 0.35–0.58). When fitting models with different numbers of subunits, we found that the median explained explainable variance increased from one to four subunits, with negligible increase when the number of subunits increased beyond four (Fig. 6). We also fitted a standard model with 12 subunits, which yielded a median explained explainable variance of 0.47 (quartiles, 0.37–0.60), only slightly greater than that with four to six subunits. When taking the maximum goodness-of-fit for each neuron across the different number of subunits fits, the median explained explainable variance became 0.49 (quartiles, 0.39–0.60). Thus, the standard CAP model captured about half of the explainable variance of the responses of the neurons. We also fitted other versions of the CAP family of models, which differed from the standard model (E-I-NL model) by allowing only excitatory subunits (E-NL model; see Materials and Methods), no nonlinear interactions between the subunits (E-I model), or only excitatory subunits without any interaction (E model). As shown in Fig. 6, the CAP family models provided overall similar fits of the shape selectivity of single MSB neurons.

**Figure 7.**
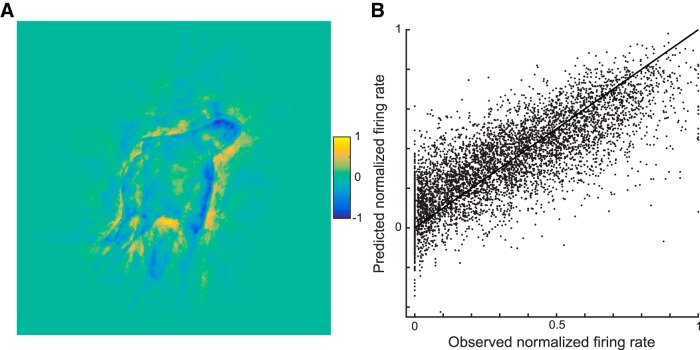
Performance of the pixel-based model. ***A***, Weights for each image pixel of the pixel-based model fitted to the responses of example neuron C65b. The weights were normalized by the maximal absolute weight. Because the shape was black and surrounded by a high (background) value in pixel intensity, the negative and positive weights imply a positive and negative contribution of the shape’s pixel, respectively, in predicting the firing rate. ***B***, Scatterplot of observed and predicted normalized firing rates to all test stimuli (not used for training the models) of the 77 neurons. Firing rate was normalized for each neuron by the maximal observed response of the neuron in the train and test data. The line corresponds to the diagonal.

The CAP family of models restricts the input of MSB neurons to local spatial filters in the orientation × curvature domain, which agrees with classic models of early visual cortical processing. However, this may be too simple a view of visual processing at levels upstream from MSB. Furthermore, CAP models without nonlinear interaction among subunits performed as well as the standard CAP model (Fig. 6). Both considerations led us to fit a simple pixel gray-level model to our data. That model consists of a linear combination of the pixel gray levels of the stimuli, without any spatial constraints on how the pixels are combined linearly. The input vector of this model consisted of the concatenated rows of the gray levels of an image. The model multiplied the gray level of each pixel value of a silhouette by a weight, and the response of the model neurons is simply the summation of these weighted values. Note that this model has no nonlinearity. For each neuron, we estimated the pixel weights with PLS regression (see Materials and Methods) using cross-validation and testing on held-out independent data. This pixel-based model produced a mean goodness-of-fit (*r*) of 0.79 for the example neuron C65b (explained explainable variance = 0.66). The estimated weights of the image pixels for that neuron, which can be read like a linear receptive field map, suggest a configuration of neighboring negative and positive weights, akin to highly local edge detectors (Fig. 7*A*). Across the 77 neurons, the median explained explainable variance (computed on independent test data) of the pixel-based model was 0.56 (quartiles, 0.45–0.68), which was well above those of the CAP model family. To illustrate the correspondence between observed and predicted test data of all 77 neurons for the model, we plotted the normalized observed and predicted responses of all test data of the 77 neurons in Fig. 7*B*. For each neuron, we first normalized both observed and predicted responses by the maximal observed response of the neuron in the train and test data. Although there is considerable scatter, the model captures the overall observed response distribution of the test data.

**Figure 8.**
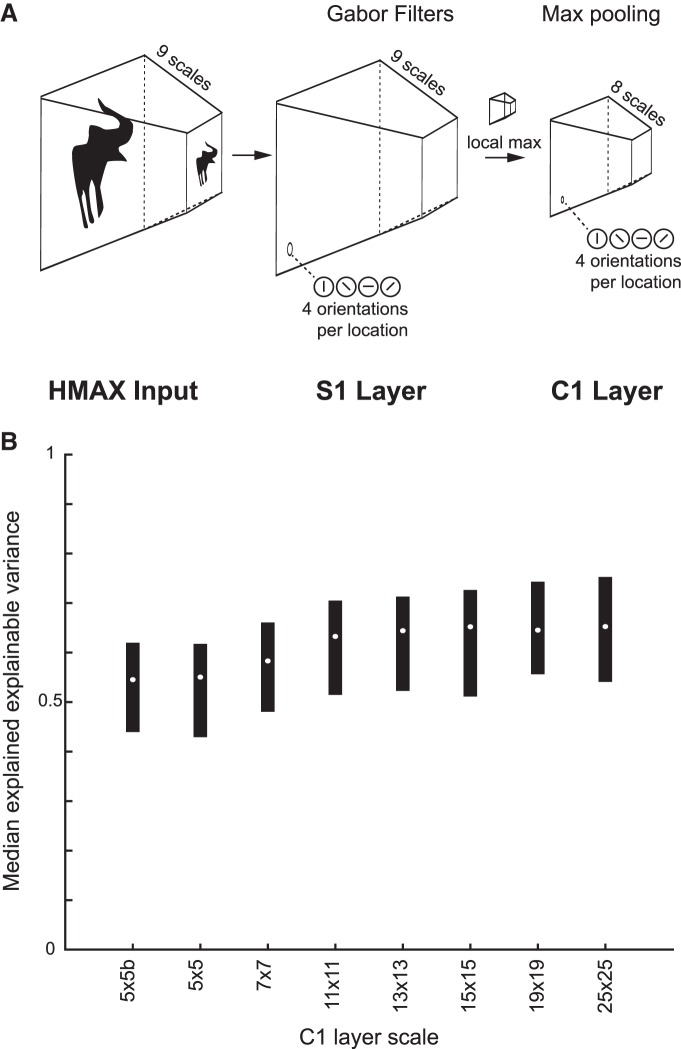
Performance of the HMAX C1 models. ***A***, Illustration of the HMAX architecture up to the C1 layer. The input image was rescaled at nine levels and then filtered by the S1 layer units at each scale by four oriented Gabors. The C1 layer performs a max pooling operation on the S1 units across location (10 × 10 S1 units) and scale (depth of 2). We applied PLS regression on the C1 layer output for each of the eight scales separately. Adapted from Mutch and Lowe (2006). ***B***, Median explained explainable variance of the pixel-based models (*n* = 77 neurons) as a function of the C1 layer scale. The bars indicate the interquartile range. The scale dimension is represented by the number of C1 units per orientation of the scale; thus, 25 × 25 corresponds to a finer scale than 5 × 5. The size of C1 layers’ scales is defined by a function (see base model of Mutch and Lowe [2006]) that specifies how the max pooling filters for each scale will span their respective inputs. The two 5 × 5 scales have the same *x* and *y* dimensions but differ in terms of the starting position of their filters’ centers, resulting in dissimilar unit activations.

Pixel-based models perform a template matching operation and as such do not capture the tolerance for position or size of the shape preference of MSB neurons (Popivanov et al., 2015). Convolutional network models with nonlinear max operations (Riesenhuber and Poggio, 1999) show some degree of position and size tolerances (see also below). Thus, in the next model, we represented the stimuli by activations of second-layer C1 units of the shallow convolution network HMAX. C1 units perform the nonlinear max operation on the output of a local set of linear orientation-selective S1 units, allowing some degree of position and size invariance of oriented features (Fig. 8A; Riesenhuber and Poggio, 1999). We used the HMAX implementation of Mutch and Lowe (2006), using eight C1 scales and four orientation filters differing in steps of 45°. We used PLS regression to predict the responses of the single units with the C1 outputs as predictor. The PLS procedure, with fivefold cross-validation and testing on held-out independent data, was identical to that used above. The median explained explainable variance, computed across the 77 neurons, increased with finer scale (Fig. 8*B*) from 0.55 to 0.65 (quartiles, 0.54–0.75). This linear combination of fine-scale C1 unit activations showed a greater goodness-of-fit compared with the linear pixel-based model. Concatenation of the activations of all eight HMAX C1 scales in one vector and using this vector as input for the PLS regression did not significantly improve the explained explainable variance (median 0.66) compared with the fine (25 × 25) C1 scale (Wilcoxon sign rank test, *p* = 0.36).

**Figure 9.**
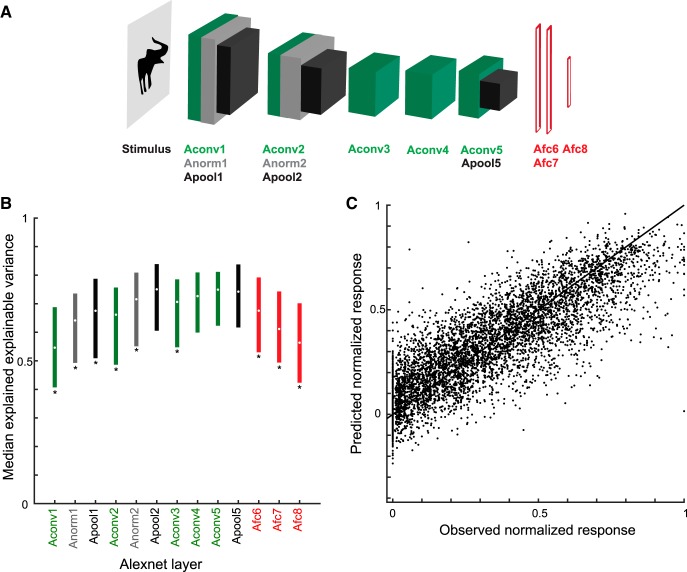
Performance of Alexnet layer models. ***A***, Architecture of Alexnet (Krizhevsky et al., 2012). Alexnet consists of eight weight layers: five convolutional layers followed by three fully connected layers (Afc6-8; red). Layers 1, 2, and 5 consist of different stages: convolution (Aconv; green), normalization (Anorm; gray), and max pooling (Apool; black). ***B***, Median explained explainable variance of Alexnet models (*n* = 77 neurons) as a function of layer. The bars indicate the interquartile range. Stars indicate layers for which the explained explainable variance differed significantly from the Apool2 stage (two-sided Wilcoxon signed rank test; false discovery rate corrected *p* < 0.05), which had the best performance. ***C***, Scatterplot of observed normalized firing rates and those predicted from Apool2. Data of all test stimuli (not used for training the model) of the 77 neurons. Same conventions as Fig. 7B.

**Figure 10.**
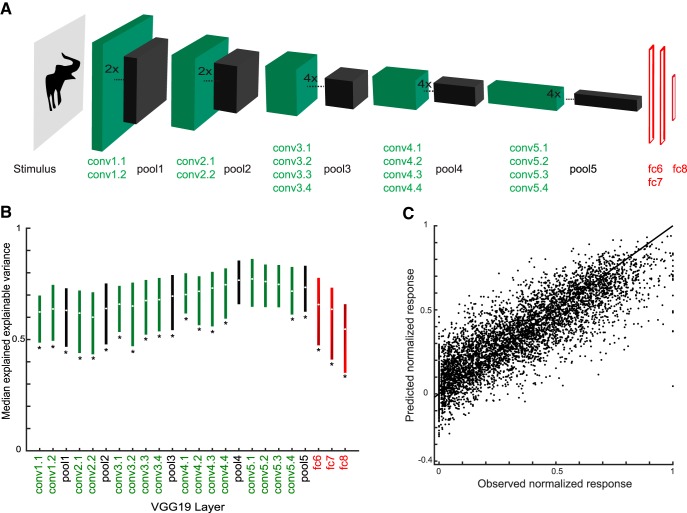
Performance of VGG-19 layer models. ***A***, Architecture of VGG-19 (Simonyan and Zisserman, 2014). VGG-19 consists of 16 convolutional layers (conv; green) followed by three fully connected layers (Afc6-8; red). In addition, it has five stages performing a max pooling operation (pool; black). Not all convolutional layers are represented by a box. ***B***, Median explained explainable variance of VGG-19 models (*n* = 77 neurons) as a function of layer. The bars indicate the interquartile range. Stars indicate layers for which the explained explainable variance differed significantly from the conv5.1 layer (two-sided Wilcoxon signed rank test; false discovery rate corrected *p* < 0.05), which had the best performance. ***C***, Scatterplot of observed normalized firing rates and those predicted from conv5.1. Data of all test stimuli (not used for training the model) of the 77 neurons. Same conventions as Fig. 7B.

The C1 units correspond to the second layer of the convolutional network architecture, after a single nonlinear operation. Because of the increase of the fits of model and neural data when using C1 activations, we asked whether fits would improve even more when considering deeper CNN layers. Thus, we turned to deep CNNs and examined the fits of each CNN layer separately. We used two deep CNNs, the eight-layer Alexnet (Krizhevsky et al., 2012) and the 19-layer VGG-19 (very) deep network of Simonyan and Zisserman (2014). Figs. 9*A* and 10*A* show the architectures of these two deep networks. Both models include a hierarchy of convolutional layers, some of which have max pooling stages and, in the case of Alexnet, normalization stages. The last convolutional layer provides then the input to the first of three fully connected layers. Both models were pretrained to classify ∼1.2 million natural images of the ILSVRC 2012 database into 1000 classes. Importantly, many of these classes included bodies and animals. The top-1 and top-5 classification error scores on the ILSVRC 2012 validation database were 29% and 10%, respectively, for VGG-19, which is better than that of the less deep Alexnet (error scores of 43% and 20%, respectively). Note that the networks were not trained with our silhouettes. We performed PLS regression to predict the responses of our single units using as predictors the activations of each layer separately. For each layer and stage, we used the same PLS regression procedure as above, with fivefold cross-validation and testing on independent data. Figs. 9*B* and 10*B* plot the median explained explainable variance for the 77 neurons as a function of the different layers and stages for both deep networks. In both networks, the best predictions were obtained with the deeper convolutional layers, with the highest median explained explainable variance being 0.77 (quartiles, 0.65–0.86; median goodness-of-fit *r* = 0.80) in the 13th convolutional layer of VGG-19 (Fig. 10*B*). The performance of the initial convolutional layers of the deep networks was similar to that of the HMAX C1 layer. However, deeper convolutional layers significantly outperformed the C1 layer of HMAX (Wilcoxon signed rank test for 25 × 25 C1 layer versus Alexnet conv5, *p* = 2.2 × 10^−5^; 25 × 25 C1 versus VGG-19 conv5.1, *p* = 1.8 × 10^−8^) and more shallow layers of the deep networks (e.g., Alexnet conv2 versus conv5, *p* = 1.8 × 10^−10^; VGG-19 conv2.1 versus conv5.1, *p* = 3.5 × 10^−10^). Note that in both deep CNNs, the fully connected layers perform relatively poorly, with VGG-19, the last (fully connected) layer, having even a lower performance (median explained explainable variance, 0.55) than the first convolutional layer (0.62). As shown by the scatterplots of the normalized observed and predicted responses, both Alexnet (Fig. 9*C*) and VGG-19 (Fig. 10*C*) deep layers predicted the differences among low, medium, and high observed responses of the neurons.

The above data show that a linear combination of units of a deep convolutional layer of deep CNNs provides an excellent model of the shape selectivity of mid-STS body patch neurons. We note that the explained explainable variance of 0.77 is only a lower bound of the real explained variance of the model, since our (split-half) response reliability metric does not take into account nonstationarity of the responses across generations. From the second generation of the adaptive stimulus sampling test onward, five stimuli per generation were repetitions of randomly selected stimuli from a previous generation (note that repetitions of the same stimuli were not used in training and testing of the models). The correlation between the responses to these stimuli presented in their first and the later generation can provide an estimate of the stationarity of the response selectivity across generations in the adaptive sampling procedure. Note that in the adaptive sampling procedure, more effective stimuli can be included in subsequent generations, which can increase cross-adaptation and thus affect overall firing rate. The inclusion of the “random” shapes and other less effective stimuli in each generation coupled with the interleaved stimulus presentation aimed to reduce such adaptation effects. Correlating presentations of the same stimulus across generations can provide a lower-bound estimate of non-stationarities, such as adaptation and long-term modulatory influences on the responses (that entered the modeling procedures). The median correlation coefficient of the responses to the first and later generation of these “control” stimuli (*r_stat_*) was 0.86 (quartiles, 0.76–0.92; *n* = 77 neurons). The correlation coefficient of *r_stat_*^2^ and the explained explainable variance of the VGG-19 conv5.1 layer was 0.35, which is significantly greater than 0 (*n* = 77; *p* = 0.002). This suggests that, indeed, our estimated explained explainable variance of the VGG-19 conv5.1 layer is still an underestimation of the real explained explainable variance of that model.

**Figure 11.**
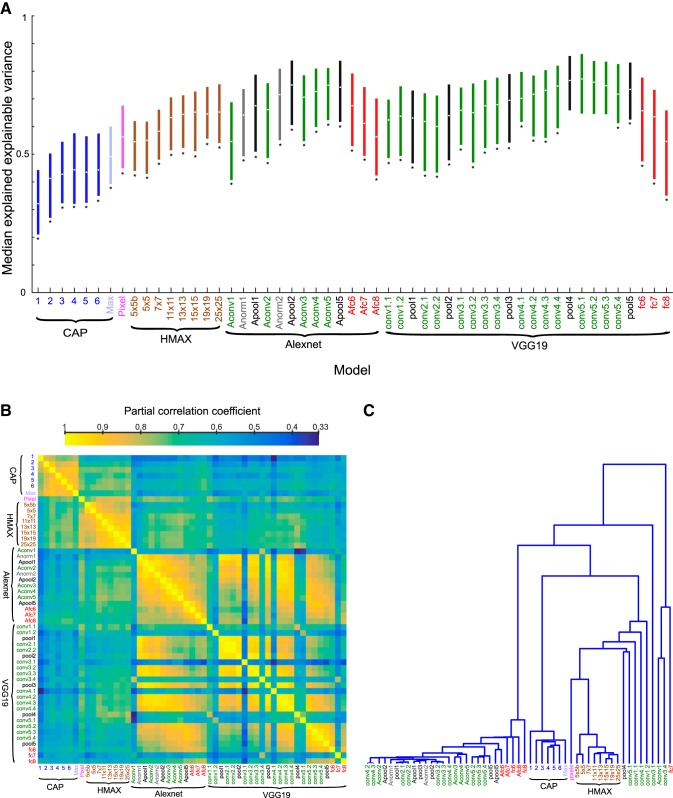
Comparison of models. ***A***, Median explained explainable variance (and interquartile range) for the E-I-NL CAP models (blue; ordered according to number of subunits; Max corresponds to the maximal explained explainable variance computed per neuron across number of subunits and model versions), the pixel-based model (pink), HMAX C1 layer (brown; ordered per scale), and the Alexnet and VGG19 layers (same color code as in Figs. 9 and 10). Stars indicate models for which the explained explainable variance differed significantly from the VGG-19 conv5.1 layer (two-sided Wilcoxon signed rank test; false discovery rate corrected *p* < 0.05), which had the best performance. ***B***, Pairwise partial correlations between the explained explainable variance of the different models, with the stationarity coefficient, *r*_stat_^2^, of the neural responses as covariate. Note that the correlation matrix is symmetric. Models are indicated using the same conventions as in ***A***. ***C***, Hierarchical clustering dendrogram of the correlation matrix of ***B***. Same conventions as in ***A***.

Fig. 11*A*produces an overview of the median explained explainable variance of the different models. The deeper convolutional layers of the deep CNNs, in particular VGG-19, provide the best fits of neural selectivity. The models marked by a star showed a significantly lower explained explainable variance than the VGG-19 conv5.1 layer model (Wilcoxon signed rank test; false discovery rate [Benjamini and Hochberg, 1995] corrected *p* < 0.05). Are the different models just noisier versions of the best model, or do different models capture different aspects of the shape selectivity? To answer this, we computed all pairwise partial correlations between the explained explainable variance of the different models, with the stationarity coefficient, *r_stat_*^2^, of the neural responses, as covariate. The partial correlation coefficients (Fig. 11*B*) were all positive, ranging from 0.33 to 0.99 (median 0.68). The correlation matrix of Fig. 11*B*was structured, with some models showing much higher correlations than others. To gain more insight into the relationship among models, we performed a hierarchical clustering analysis (single linkage algorithm) of the correlation matrix, with 1 – partial correlation as distance metric. As shown in Fig. 11*C*, many layers of both Alexnet and VGG-19 clustered together. The same holds for the CAP and the HMAX C1 models, but both these model families belonged to different clusters. The pixel model clustered with HMAX C1. The best performing VGG-19 conv5.1 layer correlated relatively well with VGG-19 pool4, which produced a similarly high fitting performance (Fig. 11*A*), but correlated less with the earlier layers and fully connected layers (Fig. 11*C*). For instance, the correlation between the VGG-19 conv5.1 layer and conv3.1 was 0.50, whereas the correlation between VGG-19 conv5.1 and pool4 was 0.76. Despite the relatively lower correlations of VGG-19 conv3.1 with the VGG-19 conv5.1 and pool4 layers, only 16/77 and 13/77 neurons showed a lower fit for the VGG-19 conv5.1 and pool4 layer, respectively, compared with the conv3.1 layer. These neurons had explained explainable variance ranging from 0.5 to 0.95 for the VGG-19 conv5.1 and pool4 layers and thus, overall, did not show a low fit for the VGG-19 conv5.1 and pool4 layers. Thus, for the large majority of neurons, the on-average worse model conv3.1 underperformed the on-average better models. This was also the case when examining models from different families that correlated poorly (CAP and HMAX versus deep CNN layers), e.g., for only one neuron, the VGG-19 conv5.1 layer underperformed the CAP model with six subunits. Therefore, it appears that the bulk of the differences in pairwise correlations reflect differences in overall fitting performance of the models, with poor models capturing the neurons’ shape selectivity only partially. This was supported by the Pearson correlation coefficient of –0.41 between the pairwise correlations of the models and the pairwise absolute difference in mean explained explainable variance of the models.

**Figure 12.**
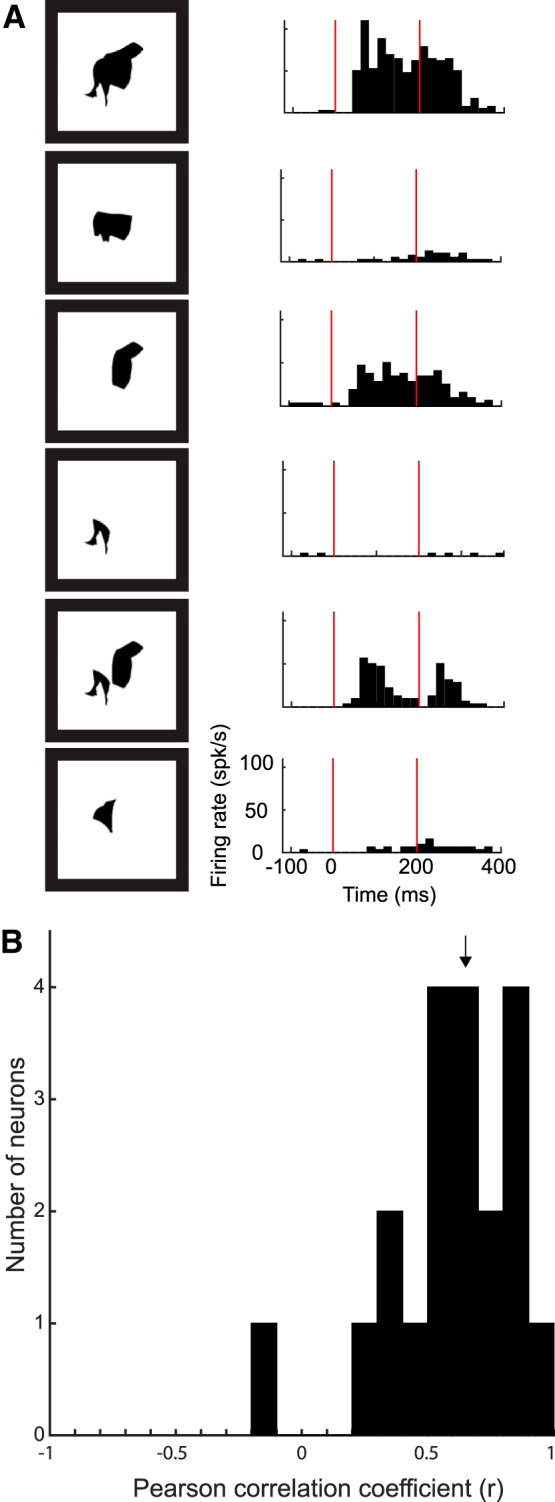
Shape decomposition test. ***A***, Example of six stimuli used in the shape decomposition test of example neuron C65b, including the top image (upper panel), and corresponding responses of the neuron. The red lines in the poststimulus time histograms indicate stimulus onsets and offsets, with 0 corresponding to stimulus onset. ***B***, Distribution of the correlation between the observed responses and those predicted by VGG-19 conv5.1 model. Data are shown for the 20 neurons tested in the shape decomposition test and for which the explained explainable variance of the VGG-19 conv5.1 model was at least 0.50 (adaptive sampling test stimuli). The median correlation is indicated with an arrow.

After the adaptive sampling test, we conducted the shape decomposition test in 27 neurons. In this test, we presented the top image of the adaptive sampling test and reduced or rotated versions of it. The reduced version consisted of parts like a limb-like feature, a head-like feature, or part of the torso. The parts were presented at the same size and at the same location as they occurred in the top image. Fig. 12*A*shows an example of six stimuli and responses of the example neuron C65b as tested in the shape decomposition test. The response of the neuron was strongly affected by removing some parts of the top image, which was typical for all neurons examined with this test. We asked whether the VGG-19 conv5.1 model would predict the responses to the stimuli of the shape decomposition test. Note that the PLS regression that estimated the unit’s weights used only stimuli of the adaptive sampling test (see Materials and Methods). To avoid any dependence between the stimuli used to train the model and the test stimuli, we predicted only the responses to the parts and rotated versions of the top image, excluding the top image. We predicted the responses to these stimuli for 20 neurons for which the explained explainable variance of the VGG-19 conv5.1 model (for the adaptive sampling test stimuli) was at least 0.50. The median correlation between the predicted and observed responses to the parts and rotated stimuli was 0.64, which was significantly greater than 0 (*n* = 20 neurons; Wilcoxon test, *p* = 1.03 × 10^−4^; Fig. 12*B*). This demonstrates that the VGG model captured a sizable part of the variability of single MSB neuron responses to different fragments of the top images.

**Figure 13.**
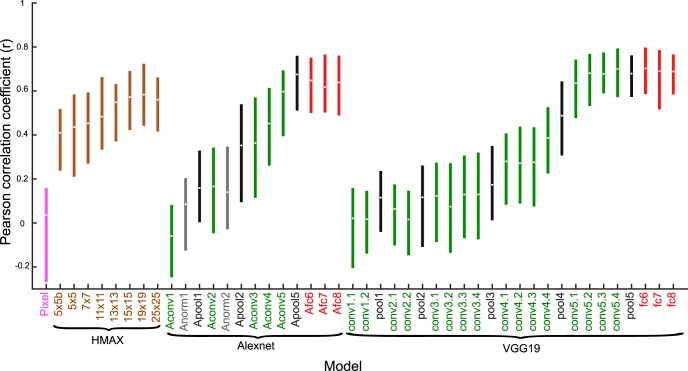
Scale tolerance of models. We computed for each neuron the Pearson correlation coefficient between the model responses to the shapes with the original and reduced scale. The medians and interquartile ranges are plotted per model using the same conventions as in .

Previous studies of MSB neurons showed that the stimulus selectivity of these neurons tolerates well changes of stimulus scale (Popivanov et al., 2015, 2016), in agreement with the well-established scale invariance of shape preference of IT neurons (Vogels and Orban, 1996; DiCarlo et al., 2012). This implies that a valid model of an MSB neuron should also show scale-tolerant stimulus selectivity. Thus, we assessed the responses of the models of each neuron to the shapes that were two times smaller than those used to train the models. This was done for all previously fitted models except the CAP models, which did not perform relatively well for the stimuli at the original scale. Then, for each neuron, we computed the Pearson correlation coefficient between the model responses to the shapes with the original and reduced scale. The median correlation coefficients expressing the scale tolerance are shown for the tested models in Fig. 13. This scale tolerance test produced further differentiation between models with similar goodness-of-fits. Although the pixel-based model produced a reasonable median explained explainable variance of 0.56, it showed no tolerance for scale variation (median *r* = 0.03). This is expected, since the pixel-based model performs a simple template matching and its output is critically dependent on the position of the pixels’ gray levels with respect to the template. Also, the initial layers of the deep CNNs showed no or little scale tolerance, whereas the scale tolerance was high in deeper CNN layers (Fig. 13). The Alexnet conv5 and VGG-19 conv5.1 layer, which had high explained explainable variance, showed asymptotic levels of scale tolerance, indicating that these models not only fit well the shape selectivity of the MSB neurons but also showed scale tolerance as MSB neurons do. The fully connected layers demonstrated levels of scale tolerance similar to those of the deepest convolutional layers, but, as we described above (Figs. 9*B* and 10*B* ), showed weaker goodness-of-fits of the shape selectivity than the deep convolutional layers.

## Discussion

We examined the shape selectivity of single neurons of the macaque body category selective patch MSB, which was defined with fMRI in the same two monkeys. Instead of measuring responses only to a random collection of arbitrary shapes, we explored the shape space locally around shapes that were shown to be responsive in an initial search test that included a fixed set of silhouettes of various object categories. This adaptive sampling procedure produced shapes that elicited stronger responses than in the search test. Many, but not all, of these top images were rated as body-like by naive human observers. The adaptive sampling procedure produced a large range of responses in each single neuron to large number of shapes, allowing quantitative modeling of the shape selectivity. We tested a range of quantitative models of shape selectivity and showed that the deeper convolutional layers of current deep neural (CNN) networks provided the best models, explaining on average close to 80% of the explainable variance of the MSB responses to the shapes. Furthermore, the deeper CNN layer units also demonstrated size tolerance of the shape selectivity and explained to a significant extent the response to shape fragments.

The purpose of the adaptive stimulus sampling procedure was to obtain a wide range of responses to a variety of shapes and not to search for the optimal shape for an MSB neuron. We believe it is impossible to determine the optimal shape for a neuron, since one cannot sample with sufficient resolution the vast shape space. One may naively expect that body patch neurons prefer shapes that are recognizable as bodies, but this was not the case for the top shapes of several of our MSB neurons. This may reflect a limited sampling of the shape space by our adaptive stimulus sampling procedure. However, nonbody shapes produced sizable responses in some MSB neurons, showing that MSB neurons can respond strongly to shapes that are not easily recognizable as bodies. The top images of the MSB neurons, although not likely identical to the optimal shapes for the neurons, are in line with previous observations showing that some MSB neurons respond well to natural images of nonbodies and sometimes even faces (Popivanov et al., 2014, 2016). The present data support our previous suggestion that MSB neurons do not respond to bodies as such but to (shape) features that happen to be part of or resemble those present in images of bodies. These features can be embedded in nonbody configurations, explaining the responses to nonbody images. This is conceptually similar to what has been observed in the face patch ML neighboring MSB. ML neurons are selective to the contrast polarity of regions of a face and respond also when such a contrast polarity relation is present in images that humans do not classify as a face (Ohayon et al., 2012). MSB and ML neurons are not “semantic” body and face detectors, respectively.

The CAP model has been applied to the shape selectivity of IT neurons that were randomly sampled from the ventral bank and lip of the STS at anterior-posterior levels that apparently included the level of MSB (Brincat and Connor, 2004). The mean goodness-of-fit of the CAP models (maximally six subunits) for the neurons in Brincat and Connor (2004) was 0.70, which is higher than our mean *r* of 0.58 for 6 subunits. The Brincat and Connor (2004) stimulus set consisted of relatively simple and highly constrained shapes with systematic manipulations of orientation and curvature, unlike our stimulus set of more complex shapes that were derived from silhouettes of natural objects and adapted to the neuron’s responses. The higher complexity of our shapes may require more than six subunits of the CAP model (the maximal subunit number used by Brincat and Connor [2004]), but increasing the number of subunits to 12 only slightly increased the goodness-of-fit (mean *r* = 0.59). A possible reason of the difference in goodness-of-fit between Brincat and Connor (2004) and our study is that because of the random sampling and wide range of locations in their study, it is likely that few if any of their neurons were from MSB. It is possible that the principles of the shape selectivity of MSB neurons differ from those of other STS neurons. In fact, there is fMRI evidence showing curvature-preferring patches along the ventral bank and lip of the STS (Op de Beeck et al., 2008; Srihasam et al., 2014; Yue et al., 2014), suggesting a heterogeneity of shape processing within the STS.

The CAP models performed similarly well when their subunits had exclusively excitatory subunits compared with mixed excitatory and inhibitory subunits. We expected that CAP models with both inhibitory and excitatory units would perform better than without excitatory units, because the stimulus selectivity of MSB neurons likely depends on both excitatory and inhibitory inputs (as other IT neurons do; Wang et al., 2000). Because CAP models with and without inhibitory units captured similar amounts of response variance, it is difficult to decide by modeling alone which of these two kind of models comes closer the neurobiological truth. Also, CAP models with or without nonlinear interactions among subunits performed similarly. Because of the higher fits we obtained with other models, we did not examine the CAP family of models further. The linear pixel-based model outperformed even the best CAP model, likely because it is not constrained by having subunits that are selective for the orientation and curvature of contour segments (as is the CAP model). However, the pixel-based models do not show size tolerance and hence cannot capture the overall stimulus selectivity of MSB neurons. CNNs with several layers that included nonlinear stages and were trained to classify a large number of natural images showed size tolerance in their deeper layers. These deep-layer CNN models produced the best fits of the models we tested, culminating in a median goodness-of-fit (*r*) equal to 0.80 for the late deep learning convolutional layers, explaining the large majority of the explainable variance of single MSB neurons’ responses to shapes.

We predicted the neuron’s response by a linear weighted combination of the outputs of the units of a CNN layer. This approach is based on the argument that one cannot expect a one-to-one mapping of the shape selectivity of a single neuron and a single unit of a model layer for the same reason that one cannot expect the same shape selectivity among units sampled in two different monkeys (Yamins and DiCarlo, 2016). However, it implies that not the CNN units but their weighted linear combination—which is one (linear) processing stage further—constitutes the model of the neurons.

Both deep CNNs showed an increase in their predictability of single MSB neuron shape selectivity with increasing layer. A similar trend has been observed when predicting macaque IT multi-unit selectivity (Yamins et al., 2014), voxel activations in human lateral occipital (LO) area (Guclu and van Gerven, 2015), and the representation similarity of macaque and human (putative) IT (Khaligh-Razavi and Kriegeskorte, 2014; Cichy et al., 2016) with layers of deep CNNs. The fact that the shape selectivity of single MSB neurons is best predicted by the deeper CNN layers suggests that these neurons respond to features that are more complex than oriented and curved contours, which are preferred more by initial CNN layers (Zeiler and Fergus, 2014; Guclu and van Gerven, 2015; Yosinski et al., 2015). Unlike in some fMRI studies that examined putative human IT (Khaligh-Razavi and Kriegeskorte, 2014; Cichy et al., 2016), the fully connected layers performed worse than the late CNN layers in predicting MSB shape selectivity. The fully connected layers are close to or at the categorization stage of the CNN and are likely strongly dependent on the specific classifications the network was trained on. The relatively poor performance of the fully connected layers suggests that single MSB neurons do not carry much categorical information, i.e. show little invariance across exemplars of the same semantic category, unlike fully connected CNN units (Yosinski et al., 2015).

We modeled 2D shape selectivity of MSB neurons because Popivanov et al. (2015) showed a sizable correspondence between their preference for the original textured and shaded images and their silhouettes. However, this correspondence was not perfect for some MSB neurons, even when taking into account response reliability. CNNs will likely be able to model texture and shading cues—these are present in the images used for training CNNs—and can be extended to encode binocular disparity cues, all of which may affect MSB responses. Bodies can be identified from distances in which surface and binocular depth cues can be poorly resolved. Indeed, bodies of different animals and their postures can typically be identified from their silhouettes, explaining the strong contribution of shape selectivity to MSB responses (Popivanov et al., 2015). Here we show that MSB shape selectivity can be modeled quite well by deep convolutional layers of CNNs that were trained to classify natural images. It is noteworthy that we could model responses to silhouettes from models that were trained with natural images. The classes the CNNs were trained on included images of bodies of animals and humans. It is not yet known whether CNNs that were not trained with body images would also model the shape selectivity of the body patch neurons. Another avenue for research is how the MSB shape selectivity and their models differ from that of the more anterior STS body patch.

## Synthesis

Reviewing Editor: Li Li, New York University Shanghai Decisions are customarily a result of the Reviewing Editor and the peer reviewers coming together and discussing their recommendations until a consensus is reached. When revisions are invited, a fact-based synthesis statement explaining their decision and outlining what is needed to prepare a revision will be listed below. The following reviewer(s) agreed to reveal their identity: Bevil Conway, David Sheinberg

Both reviewers agree that the ms is in good shape now and can be accepted for publication. R2 has some comments that should be addressed before the ms is formally accepted. Accordingly, please submit a revision and I will make the final decision without going through any more external review. Please see below for the detailed comments:

R1:

This is a revision of a manuscript I reviewed at J Neuroscience. The authors have addressed my concerns and the paper makes a useful contribution to the field.

R2:

This version of the Kalfas et al. manuscript is very similar to that previously reviewed. As indicated in my original review, I think the two building blocks of the paper used to explore shape selectivity in MSB - the adaptive stimulus generation approach and the model comparison approach - are appropriate and well described. The authors have modified the Introduction to provide a bit more in the way of rationale for their study (especially the last paragraph of the Introduction).

My reservations regarding the major impact of the paper do remain, though. The results of the stimulus optimization and the model fitting lead to fairly unconstrained conclusions - 1) that shape drives these neurons and that the shape doesn't necessarily always look like a body, and 2) that deep CNNs do a good job of predicting responses to untrained images if higher layers beneath the fully connected layers are probed. These are interesting data and worth publishing. I was hoping for something a bit more “significant”, for example, in the Significance statement. As it is, it reads mostly like a description of the methods. The disconnect from the “semantics” of body detectors (as presented in the Discussion) could at least be included here.

One direction the authors could at least consider is the dynamics of the response. The whole analysis (I think) is based on mean rates between 50ms-250ms following stimulus onset. Did the authors explore the timing at which goodness of fit reached its maximum and did it differ across the response period? How this area fits within the visual processing stream might be elucidated by information about the timing of information about shape and whether it appears to be slightly later than, for example, shape responses in area TE or in more anterior regions of the STS.

As the authors have already made a couple of round of revisions, and based on their responses to commends, I think the paper itself is basically in good shape.
